# A Soil Monitoring Law for Europe

**DOI:** 10.1002/gch2.202400336

**Published:** 2025-02-25

**Authors:** Panos Panagos, Arwyn Jones, Emanuele Lugato, Cristiano Ballabio

**Affiliations:** ^1^ European Commission Joint Research Centre (JRC) Ispra Italy

**Keywords:** land degradation, policy support, soil health, soil mission, soil monitoring

## Abstract

Over 60% of European soils are unhealthy according to the Soil Mission board estimates and the indicators presented in the European Union (EU) Soil degradation dashboard. The situation may worsen if no policy interventions are taken. The unsustainable use of natural resources, in particular the degradation of soils, precipitates biodiversity loss, exacerbated by the climate crisis. In particular, in the EU alone, soil degradation costs over €50 billion per year due to the loss of essential services they provide and to the impact on human health. Here a more precise estimation of the soil degradation cost related to a set of soil degradation processes, ranging between €40.9 and 72.7 billion per year is presented. This newly updated estimate compared to the Impact assessment of the Soil Monitoring Law takes into account the costs of soil erosion, contamination, phosphorus losses, soil carbon losses, nitrogen losses, soil compaction, and soil sealing. However, this estimation might double if it is added to the costs of soil biodiversity loss, floods, droughts, off‐site effects of soil erosion, and health consequences of soil contamination. Therefore, further research is needed to address this knowledge gap and estimate the missing costs. Soil degradation is a critical issue with transboundary implications that requires urgent attention and action at the EU level. The costs of soil degradation are substantial, both in terms of environmental impacts and economic consequences, highlighting the importance of investing in sustainable soil management practices and a harmonized EU soil monitoring system. By addressing soil degradation through the proposed Soil Monitoring Law, investing significant amounts for research and innovation in the Soil Mission, and promoting international cooperation, the EU can take solid steps toward protecting its soil resources and achieving a sustainable future for all.

## Graphical abstract

Over 60% of European soils are unhealthy, costing the European Union €40.9–72.7 billion annually. This figure can rise to €140 billion if all degradation processes are included. Soil degradation worsens biodiversity loss and the climate crisis, highlighting the need for urgent policy intervention at the EU level, such as the proposed Soil Monitoring Law, and sustainable soil management practices to protect soil resources and achieve a sustainable future.

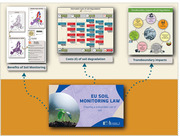

## 1 Background

Healthy soils produce food, increase our resilience to climate change, support ecosystems, and mitigate the impact of extreme weather conditions that cause droughts and floods. In the proposal for a Soil Monitoring Law, soil health is defined as “*the physical, chemical, and biological condition of the soil determining its capacity to function as a vital living system and to provide ecosystem services*” (SML, 2023).

On July 5, 2023, the European Commission tabled a proposal for a soil monitoring and resilience directive, laying down measures for monitoring and assessing soil health, based on a common definition of what constitutes healthy soils, for managing soils sustainably, and for restoring contaminated sites. On April 15, 2024, the European Parliament adopted its position on the Commission's proposal for a Soil Monitoring Law (SML), the first‐ever dedicated piece of EU legislation on soils. On June 17, 2024, the European Council gave a positive vote and underlined the importance of soil monitoring which also provides guidelines for sustainable soil management. The next step after the Council decision allows its rotating presidency to start discussion with the European Parliament on the final shape of the text. Within five years of the Directive's entry into force, Member States   will define their plans for sustainable soil management practices, in line with the EU targets.

The Soil Monitoring Law is essential due to the widespread and cross‐border nature of soil degradation, as well as the significant risks that degradation poses to the environment, economy, and society. EU‐level coordination is necessary to address the urgent issue of soil degradation and improve soil health by 2050. This draft law envisions a harmonized EU soil monitoring system that will gather data to enhance our understanding of land degradation and support land managers, owners, and society in improving soil health. The actions taken by individual Member States have, so far, proven not sufficient to reverse the current trends of land degradation that continue to increase.^[^ ^1^ ^]^ According to the European Court of Auditors, the current policy framework for soil protection in the EU is not sufficient to halt desertification and soil degradation.^[^ ^2^ ^]^ Reaching similar conclusions, the European Commission states that actions taken to address the issue have not been sufficient to reverse the current trends.^[^ ^3^ ^]^

At the international level, the EU made commitments to fight land and soil degradation in the context of the United Nations (UN) Convention to Combat Desertification (UNCCD), Framework Convention on Climate Change (UNFCCC), and Convention on Biological Diversity (UNCBD). The Sustainable Development Goals (SDGs) as part of the 2030 UN agenda, provide a roadmap for a sustainable world, including 17 goals that are translated into consistent targets. The “Land Degradation Neutrality” (target 15.3) is among those targets and the EU has committed to take action for achieving land degradation neutrality by 2030.^[^ ^1^ ^]^ This goal aims to combat desertification, restore degraded land and soil, including land affected by desertification, drought, and floods, and strive to achieve a land degradation‐neutral world by 2030.

The objective of this manuscript is to present important insights into the proposed Soil Monitoring Law, with a focus on the benefits of harmonized soil monitoring. Additionally, the manuscript compiles recent estimates of the costs of soil degradation in the European Union.

## 2 The Need for EU Intervention in Soil Protection

The existing EU environmental legislation does not target explicitly soil degradation and soil health. Rather, there are several legislations that marginally refer to some aspects of soil degradation without however addressing it satisfactorily. The EU policy agenda addresses soils in a fragmented way,^[^ ^4^ ^]^ considering either the agricultural perspective (e.g., Common Agricultural Policy, Nitrates Directive) or from a more environmentally oriented one (e.g., NATURE 2000, Water Framework Directive, Sewage Sludge, etc.). For example, the Common Agricultural Policy (CAP) address soil protection in agricultural lands, which is threatened by the intensification of agriculture. In addition, few countries have national laws on soil protection which address specific threats for soil degradation. Therefore, a holistic soil protection approach was missing in the EU to address soil protection in all lands. Compared to a decade ago when soil protection was scattered among a few agro‐environmental policies,^[^ ^5^ ^]^ now soil protection is a cross‐cutting issue in the EU policy agenda due to the pivotal role of the Green Deal.^[^ ^6^ ^]^

The lack of a comprehensive policy framework for soil protection within the EU hinders the effectiveness of existing measures. With a significant portion of the EU soils (>60%) already deemed unhealthy, urgent action is required to safeguard food security and biodiversity and mitigate vulnerability to climate change.^[^ ^7^ ^]^

The EU has previously taken legislative actions impacting soil health through various policies, such as those concerning agriculture (Common Agricultural Policy), water (Water Framework Directive), climate (LULUCF regulation), and industry (Industrial Emissions Directive). However, these efforts have not been adequate to address the scale of soil degradation. Additional EU intervention is essential to complement existing measures and bridge policy gaps comprehensively. The subsidiarity principle, which allows for varying degrees of flexibility for Member States alongside EU intervention intensities, is crucial in addressing the cross‐border aspects of the soil degradation issue.

To protect Europe's soils effectively, a holistic and integrated approach is needed, considering the multifaceted challenges posed by soil degradation. By developing policy options that respect the subsidiarity principle, the EU can work toward achieving the vision of healthy soils and sustainable land management practices by 2050.

## 3 Costs of Soil Degradation

The costs of soil degradation are estimated roughly to an amount of >€50 billion annually according to the Impact Assessment (IA) of the Soil Monitoring Law proposal.^[^ ^8^ ^]^ This figure is based on the 2006 Soil Thematic Strategy estimates of soil degradation costs, which were ≈€38 billion annually.^[^ ^9^ ^]^ The past estimates have been actualized and updated with additional knowledge available during the period 2006–2023. However, lack of relevant economic studies has resulted in a partial knowledge about the costs of soil degradation in the EU.

**Figure 1:**
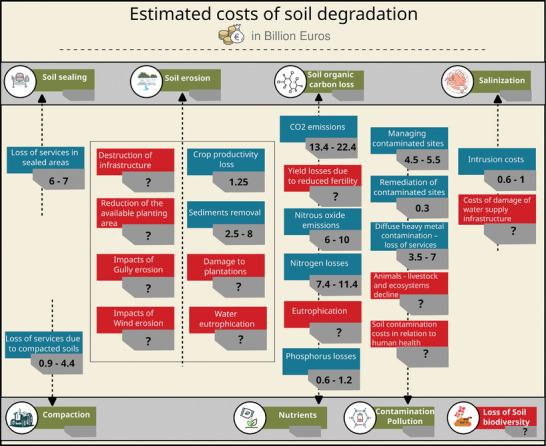
Estimated costs of soil degradation and missing costs (red).

For instance, soil erosion has onsite (land productivity loss, yield losses, nutrient losses, plant losses, etc.) and off‐site effects (increased sedimentation in rivers and lakes and the resulting potential destruction of infrastructures, loss of biodiversity and eutrophication, etc.) (**Figure** **1**). Regarding the on‐site effects, the 12 million hectares of agricultural areas in the EU that suffer from severe erosion, are estimated to lose ≈0.43% of their potential crop productivity annually. The annual cost of this loss in agricultural productivity is estimated at ≈€1.25 billion.^[^ ^10^ ^]^ The EU Soil Observatory (EUSO) working group on soil erosion made a comprehensive assessment of the costs of sediment removal from EU catchments due to water erosion to be ≈€2.5 billion. Considering the sediment delivered through all soil loss processes (gullies, landslides, quarrying, among others) and extrapolating to measured reservoir capacity losses, the sediment accumulation in the circa 5000 EU large reservoirs exceeds 1 billion m^3^ with a potential cost of removal ranging between €5 and 8 billion annually.^[^ ^11^ ^]^ Those estimates provide additional knowledge compared to the IA of the Soil Monitoring Law as they took place in 2023.

An integrated assessment of nutrient losses with sediments^[^ ^12^ ^]^ estimated that current phosphorus displacement in the EU‐27+UK due to erosion was ≈374 000 tons, of which ≈97 000 tons end up in river basins and sea outlets. The cost of Diammonium phosphate (DAP) (the most common phosphate fertilizer) has varied widely over time, particularly over the past two years and we adopted a range of prices from € 308 to 622 per ton (averaging 2013–2020 and 2021–2022 prices respectively. Then, we estimated that the cost of phosphate loss in agricultural soils due to water erosion is between €575 million and 1.2 billion annually (accounting for the total phosphate content of one ton of DAP is ≈20%).

We also estimated the costs associated with nitrogen losses using the data from the EU biogeochemical modeling framework.^[^ ^13^ ^]^ Since nitrogen is lost in different forms, we gave first‐order approximation estimates as emissions cost, for direct soil N_2_O losses and, as fertilizer substitution cost, for the inefficient fraction of elements not utilized by plants (leaching and erosion).

Considering the estimated soil N_2_O emissions of ≈103 Mt CO_2_ eq. yr^−1^ (247 Kt of N)^[^ ^14^ ^]^ and a mean CO_2_ price between 60–100 € per ton, the associated annual emission cost would be of €6–10 billion. In addition, leaching and erosion nitrogen losses sum up to 4400 Kt of N, which corresponds to €1.4 billion at the current average nitrogen fertilizer price of 320 € per ton (https://agridata.ec.europa.eu/extensions/DashboardFertiliser/FertiliserPrices.html). The last value may be slightly overestimated if we consider that even the best management on nitrogen at the EU level cannot likely reach 100% efficiency (in other words it would be impossible to have no losses from nitrogen application). This estimate does not consider the indirect environmental costs, which may occur from nitrogen losses to the water system (e.g., eutrophication).

Globally, the EU wide is the second largest emitter of greenhouse gases (GHG) from drained peatlands (220 Mt CO_2_eq yr^−1^),^[^ ^15^, ^16^, ^17^ ^]^ Regarding agricultural soils, a recent study using the topsoil LUCAS survey, has estimated a loss of 255 Mt CO_2_eq in 9 years (2009–2018)^[^ ^18^ ^]^ which is ≈28.5 Mt CO_2_eq yr^−1^. Focusing just on the EU27 (without the UK), the 223.5 Mt CO_2_eq annual emissions (mineral soils and peatlands), can be estimated to cost ≈€13.4 to 22.4 billion (using a mean cost of CO_2_ eq. between €60–100 per ton). Also, this is a new estimate which is an output of the 2024 activities in the EU Soil Observatory adding new knowledge in the costs of soil degradation.

For soil compaction, the costs may include the additional tractor (fuel) power required for tillage operations in areas affected by soil compaction.^[^ ^19^ ^]^ Soil compaction may also affect losses of soil nutrients (hence, increased fertilization) leached (through runoff) due to reduced soil infiltration.^[^ ^20^ ^]^ Assuming those costs, the soil compaction is estimated to cost ≈140 € ha^−1^. For the 6.4 million ha with high packing density (>1.75 g m^−3^) in the EU,^[^ ^21^ ^]^ the cost is ≈0.9 billion € per year. If we focus only on the 108 million ha of arable lands in the EU, 58% (62 million ha) has a medium packing density of 1.4–1.75 g m^−3^ which implies certain costs for farmers. Assigning half of the cost implied in highly compacted soils (70 € ha^−1^), the costs of compaction in arable lands may rise to 4.4 billion € annually. Another hidden cost that is often neglected is the irreversible soil degradation caused by subsoil compaction.

Salinity can cause crop yield losses as crops may lose their ability to extract water and nutrients from saline soils.^[^ ^22^ ^]^ In the USA, the saltwater intrusion into the coastal ground‐ and surface water results in loss of agricultural income to ≈€0.1–0.3 billion for the 9% of USA farmlands^[^ ^23^ ^]^ which can be of ≈1–3 billion of the whole country. Since in the EU, the farmland area is half compared to the USA and salinity is less intensive, we can assume a cost of €0.5–1.5 billion annually. However, salinity is determined by irrigation in agricultural activities which will further accelerate due to climate change in semi‐arid areas of the Mediterranean basin.^[^ ^24^ ^]^

For soil sealing, the total artificial surfaces in the European Union accounted for ≈5.6% of the total land area (2018 data). In absolute terms, this equates to ≈23.7 million ha (EUROSTAT). There is a high uncertainty on ecosystem services value lost due to soil sealing and derivative effects (floods). However, some rough estimates show that on average each hectare of artificial land is a loss of €300 in ecosystem services losses.^[^ ^25^ ^]^ Therefore, the total cost of ecosystem services lost due to all artificial lands in the EU is ≈€7.1 billion annually.

In a study estimating the cost of de‐sealing, it was proposed that the costs of removing soil sealing materials alone for ≈16–20 € m^−2^, including recycling of material.^[^ ^26^ ^]^ Therefore, the cost of removing a hectare of sealed material could be ≈€160 000–200 000. Considering that each year, in the EU, we are sealing >39 000 ha, the potential cost of restoration (de‐sealing degraded artificial lands) of such an area would be €6.2–7.8 billion annually.

Based on the impact assessment of the 2006 proposed European Soil Framework directive, the estimated cost of managing contaminated sites ranges from €2.4 to 17.3 billion. In the 2012 data collection on contaminated sites, 11 countries (23% of the population) reported that the mean cost per management of contaminated sites is ≈10.7–12 € per capita. Thus, the management of contaminated sites in the EU could be ≈€4.5–5.5 billion per year.^[^ ^27^ ^]^ However, the European Environmental Agency states that the remediation of each contaminated site may cost ≈€0.1–1 million,^[^ ^28^ ^]^ resulting in an estimated €0.3–3 billion per year to remediate an average of 3000 sites in the EU.^[^ ^29^ ^]^

For diffuse soil contamination, the current Impact Assessment of the Soil Monitoring Law^[^ ^8^ ^]^ proposed a very large range of costs varying from €3–292 billion. Based on the EU Soil degradation dashboard, the heavy metals contamination (Arsenic, Cadmium, Copper, Mercury, and Zinc) is affecting 8.3% of EU land or about to 34.4 million ha.^[^ ^30^ ^]^ Following the same approach as in the soil sealing case, we make the assumption that a contaminated land implies a cost of 100–200 € ha^−1^ for the loss of ecosystem services (compared to 300€ ha^−1^ of soil sealing).^[^ ^25^ ^]^ Soil contamination can lead to reduced crop yields or make crops unfit for human consumption due to contaminants like heavy metals.^[^ ^31^ ^]^ Contaminated soils can impair the ability of soil to filter and retain water^[^ ^32^ ^]^ and soil contamination can harm or kill soil organisms, reducing biodiversity and the overall health of the ecosystem.^[^ ^33^ ^]^ Therefore, the diffuse contamination costs will be at in the range of €3.5–7 billion as only from an ecosystem services loss perspective. The total cost of diffuse contamination is much higher as we should add costs of risk of contaminating other resources (water, air), increased healthcare costs due to illness and side effects provoked to humans and livestock, treatments, and food safety costs.

Summing up all those costs, we estimate a total annual cost of soil degradation in the range of €40.9–72.7 billion (**Table** **1**) which is partial as many important costs cannot be estimated due to lack of data and knowledge gaps. Among the costs that are missing are the cost of soil biodiversity loss, floods and droughts, landslides, off‐site effects of soil erosion, the subsoil compaction, and consequences of soil contamination in human health and livestock (Figure 1). Therefore, with most conservative estimations the annual cost of soil degradation is at least in the range of €80–140 billion in the EU. This cost is much higher compared to the estimated cost in the IA of the Soil Monitoring Law (€50 billion) as we have included new estimations for degradation processes.

**Table 1:** Estimated costs of soil degradation in the EU.

Soil degradation process	Detailed loss and estimation cost	Minimum estimate (billion €)	Maximum estimate (billion €)
Soil erosion	Crop productivity loss	1.3	1.3
	Sediments removal	2.5	8
Soil organic carbon loss	CO_2_ emissions	13.4	22.4
Contamination / Pollution	Managing contaminated sites	4.5	5.5
	Remediation of contaminated sites	0.3	3
	Diffuse heavy metal contamination–loss of services	3.5	7
Nutrients	N_2_O emissions	6	10
	Nitrogen losses	1.4	1.4
	Phosphorus losses	0.6	1.2
Compaction	Loss of services due to compacted soils	0.9	4.4
Salinity	Intrusion costs	0.5	1.5
Soil sealing	Loss of services in sealed areas	6	7
TOTAL		40.9	72.7

The staggering cost of soil degradation underscores the urgent need for effective solutions to mitigate its impact. In this context, implementing a robust soil monitoring system becomes indispensable. Such a system not only provides critical insights into the progress of efforts to improve soil health but also enhances the effectiveness of measures aimed at reducing soil degradation. By continuously tracking changes and evaluating outcomes, a monitoring framework ensures that resources are directed toward the most impactful strategies, ultimately contributing to the reduction of degradation‐related costs and fostering long‐term sustainability.

## 4 Establishing a Harmonized EU Soil Monitoring System: A Foundation for Sustainable Soil Management

Establishing a harmonized EU monitoring soil system to build a robust knowledge base is crucial to inform and support the land owners, farmers, soil managers, and policymakers. Indeed, implementing adequate and effective measures to promote healthy soils necessitates data, information, and knowledge, particularly to accommodate the significant variability in soil types, climatic conditions, and land uses. Consequently, the insights gleaned from sustainable soil management practices on the ground, inform and assist in refining monitoring and governance mechanisms.

For instance, implementing a soil health monitoring system would align with the requirements for monitoring and reporting soil organic carbon as outlined in the updated Land Use, Land‐Use Change and Forestry (LULUCF) Regulation and the Carbon Removal Certification Framework (CRCF).^[^ ^34^ ^]^ Currently, there are serious losses in croplands and gains in grasslands unreported in the EU LULUCF framework.^[^ ^35^ ^]^ In addition, within the proposed legislation for carbon faming certification, there is a need for both accurate metrics of change in soil organic carbon and advanced soil monitoring.^[^ ^34^ ^]^ This would also support the necessity of monitoring soil organic carbon stocks in croplands as stipulated in the Nature Restoration Law.^[^ ^36^ ^]^

An enhanced pan‐European soil monitoring scheme will be a major gain for farmers to make better decisions for crop selection, fertilization, irrigation, and water management.^[^ ^37^, ^38^ ^]^ Regular monitoring helps identifying nutrient deficiencies or imbalances in the soil.^[^ ^39^ ^]^ Therefore, an appropriate amount of nutrient inputs could be applied based on the specific needs of the cultivated crops and the pedo‐climatic conditions.^[^ ^40^ ^]^ This targeted approach ensures that crops receive the necessary nutrients for optimal growth, which can lead to higher yields while avoiding excess nutrient load.^[^ ^41^ ^]^ Soil monitoring can help farmers to understand the water‐holding capacity and infiltration rate of their soil, which is essential for managing irrigation needs.^[^ ^42^ ^]^ By applying the right amount of water at the right time, farmers can optimize crop growth and prevent water stress and at the same time avoid exhausting water resources. This comes together with the proper selection of crops (based on the pedo‐climatic conditions) and the application of best soil management practices.

By closely monitoring soil conditions, we can detect and prevent soil degradation that can be manifested as erosion, biodiversity loss, carbon and nutrient fluxes, contamination, and their combinations. This helps maintaining a healthy ecosystem and a toxic‐free environment. As an example, a prone to soil erosion field would need to implement certain management practices such as reduced tillage, cover crops, mulching, or crop rotation. Other important benefits of a harmonized monitoring system include disaster prevention (e.g., floods, landslides, and droughts), climate change mitigation, and better resource management including nutrient inputs.

The SML includes some innovative concepts for legislation, as it sets a common statistical framework to perform soil monitoring at the EU scale. The sampling strategy as proposed in the SML will gather data on physico‐chemical and biological soil properties with a stratified method that ensures representativeness and minimizes costs.^[^ ^30^ ^]^ This will avoid the inevitable biases that could arise from Member States implementing different monitoring schemes using different spatial sampling designs. Additionally, the law sets a target for the number of samples to include in the survey by setting a target for the coefficient of variation, for the soil properties to be estimated. This target will be reached by optimizing the number and the location of the sampling points, thus ensuring a harmonized framework among MS. The physico‐chemical and biological soil properties that will be analyzed are included in the annexes of the SML.^[^ ^8^ ^]^

**Figure 2:**
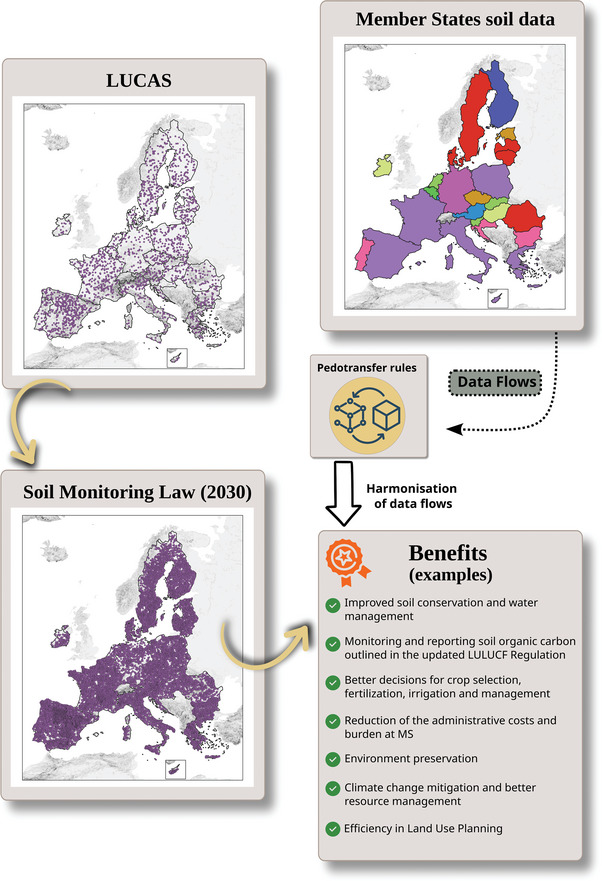
The benefits of a harmonized Soil Information system.

The SML will not drastically increase the burden for monitoring the state of soils in the EU. The European Commission will support the Member States (MS) by reinforcing the current EU Sampling program LUCAS topsoil.^[^ ^43^ ^]^ This survey was already repeated four times in the past, with ≈20 000 samples analyzed per campaign (32 000 in the last campaign of 2022). The EC plan is to extend LUCAS and increase the number of sampled points to better monitor soils in the EU (**Figure** **2**).

Before having the proposal of the SML, there was a lack of agreement on what a healthy soil is and which indicators, and which thresholds should be used for each soil type, ecosystem, and climatic zone. Consequently, there are many methodologies in place to assess soil health indicators. These methods can vary from field sampling methodologies, different depths, laboratory analysis, and targeted indicators and thresholds. Moreover, MS with long‐standing soil monitoring systems prefer to keep existing procedures, for economic reasons or to safeguard long‐term datasets.

A key aspect of harmonization of soil data, and consequent comparability at EU level and the possibility for integrating national and LUCAS datasets, is the development of “transfer function” between different soil depths, measurement protocols, and sampling methods(Figure 2). For all these reasons, transfer functions are needed to facilitate a smooth transition to a harmonized soil health assessment in the EU. Some knowledge on transfer functions is already established for various soil physical and chemical properties. However, existing knowledge does not cover the entire combination of laboratorial and field methodologies available in the EU for all the soil descriptors present in the Soil Monitoring Law. Moreover, existing pedo‐transfer functions (PTF) are often optimized on datasets limited in their geographical scope, covering a limited range of environmental conditions that are not representative of the soil variability within the EU. Therefore, efforts and research to develop “transfer function” would contribute to a consolidated and harmonized Soil Monitoring system in the EU. Such a system will be of mutual gain for both the regional, national, and EU policy makers to promote sustainable land use and the best protection of soil resources. The standardization and the integration of national data with EU LUCAS soil data will reduce the administrative costs and burden at MS to fulfill different reporting obligations in relation to LULUCF, Common Agricultural Policy (CAP), Nature Restoration Law, and other agro‐environmental policies.

A harmonized soil monitoring system is essential for detecting and addressing the transboundary impacts of soil degradation. By establishing standardized methodologies and common data collection protocols, such a system ensures unbiased and comparable assessments of soil conditions across regions and nations. This uniform approach enables the identification of degradation trends and their cascading effects on ecosystem services that extend beyond political boundaries. Moreover, it fosters international collaboration by providing a shared, credible foundation for policy‐making, reinforcing efforts to tackle soil degradation as a global challenge, and highlighting the interconnected role of soils in nutrient, carbon, and water cycles that underpin global environmental stability.

## 5 Transboundary Impacts of Soil Degradation

Soil degradation is a challenge that has implications beyond national borders and can impact the provision of ecosystem services in the EU and other countries. Soil degradation is sometimes perceived as a local issue, with its transboundary effects being underestimated. Healthy soils are essential in addressing global societal challenges, as they play a critical role in nutrient, carbon, and water cycles, which are not constrained by physical or political boundaries.

The impact of soil health on carbon and nitrogen emissions cannot be overstated. It is therefore imperative that effective measures are put in place to tackle soil degradation in all EU countries. Failure to do so in one country can have negative consequences on climate change mitigation and adaptation efforts in other Member States, as well as the EU's goal of achieving climate neutrality by 2050. It is worth noting that mineral soils under cropland lose ≈7.4 million tons of carbon annually,^[^ ^18^ ^]^ while peatland drainage in Europe alone accounts for ≈5% of total EU greenhouse gas emissions.^[^ ^44^ ^]^

It has been observed that certain management practices, such as intensive agriculture, unsustainable forest management, and peatland drainage may have an impact on soil degradation, leading to the loss of soil organic matter, acidification, salinization, compaction, and erosion. This, in turn, may increase the vulnerability of soils across borders. As an example, floods due to extreme weather events (as the 2024 events in Valencia (Spain) and Emilia Romagna (Italy) and the 2023 flood in Thessaly (Greece)) may make an entire region with high soil degradation vulnerable to natural disasters.^[^ ^45^ ^]^ Therefore, the role of soils is recognized as a key ecosystem component, in the attempt to regulate critical hazards such as floods, droughts, landslides, and dust storms.^[^ ^46^ ^]^

It has been estimated that the costs of erosion that occur off‐site (loss of biodiversity, decreased food supply, sedimentation, etc.) are significantly higher than those that occur on‐site. For example, soil particles are eroded by water, they are transported downstream and across borders through the soil‐sediment‐water system. This can lead to an increase in turbidity, a reduction in water quality, and an increase in sedimentation and water treatment costs. As sediment contamination and siltation are among the major issues impacting port operations and management, port authorities may spend significant amount of money on remediation.^[^ ^47^ ^]^ Many ports as the ones of Rotterdam, Hamburg, Dublin, and Antwerp undertake significant dredging operations each year to maintain proper water depths and easy navigability.^[^ ^48^ ^]^ A considerable proportion of the sediment removed in the North European ports is caused by sediments transferred by the Rhine and can be attributed to unsustainable soil erosion upstream. ≈25% of the ≈100 transboundary river basins in the EU have identified soil erosion as a significant issue, primarily due to agricultural practices. Therefore, it is crucial for all countries to work together to address this issue and find sustainable solutions. An integrated EU management of water and sediments is requested.^[^ ^49^ ^]^ It is important to note that soil erosion in one country can result in sediments being washed away and blocking dams or damaging infrastructure, such as harbors, and dams in other countries.^[^ ^50^ ^]^ It is essential to tackle this issue through erosion prevention and sustainable soil management in the country of origin, which can be a cost‐efficient solution.

Excessive nutrient use and runoff from soils is a matter of concern as it can result in cross‐border eutrophication of water bodies and seas. Eutrophication can be understood as a misbalance between in‐ and outputs (sources and sinks) of nutrients, leading to increased accumulation of organic matter in aquatic systems.^[^ ^51^ ^]^ As an example, the oversupply of nutrients in agricultural land in the countries surrounding the Baltic Sea is a significant environmental pressure on groundwater aquifers and the marine ecosystem.^[^ ^52^ ^]^ Even reducing its nitrogen emission, Europe has been identified as a significant contributor to global nitrogen pollution, as it exports high levels of nitrogen through rivers to coastal waters.^[^ ^53^ ^]^

Soil particles and adsorbed chemicals can be transported over long distances across borders due to wind erosion. For instance, glyphosate and aminomethylphosphonic acid (AMPA), a glyphosate metabolite, are among the chemicals that can be transported by wind.^[^ ^54^ ^]^ Wind erosion affects both the semi‐arid areas of the Mediterranean region that cross borders and the temperate climate areas of northern and central European countries.^[^ ^55^ ^]^ Furthermore, the deposition of heavy metals caused by air and water pollution originated from anthropogenic industrial activities can have negative effects on chemical and biological processes in soils.

As a transboundary example, we focus on the Rhine River, which is an important waterway that spans several European countries, including Switzerland, France, Germany, and the Netherlands. Contaminated sediments (containing heavy metals, PCBs, and other pollutants) are transported downstream due to natural processes like erosion and sedimentation, as well as human activities, such as dredging.^[^ ^56^ ^]^ The consumption of contaminated fish and shellfish from the affected water bodies can pose a risk to human health as the example of high levels of mercury in fish.^[^ ^57^ ^]^ Another side effect can be in the economy of a region, as the declining fish populations due to contaminated transferred sediments can impact commercial fishing and aquaculture industries, resulting in job losses and reduced income.^[^ ^58^ ^]^

Contaminants introduced to soil have the potential to leach into groundwater, surface, marine, and coastal waters, which can affect the quality of drinking and bathing water. This can ultimately result in contamination of the sea. Additionally, soil contamination can pollute transboundary aquifers through infiltration and runoff. Examples of transboundary contamination can be observed in the Campine area (cross‐border Flanders and the Netherlands), Werra River basin (Germany and Czechia), and Danube River basin. Another example of cross‐border effects is the large‐scale PFAS contamination caused by a chemical producer in Antwerp, which is mobile and crosses the border with the Netherlands.^[^ ^59^ ^]^

Soil contamination can pose a potential threat to food safety both within Europe and globally. Agricultural soil contamination has the potential to result in transboundary risks, leading to food contamination that can circulate freely within the EU internal market. For example, a significant number of Europeans, including children, may be exposed to cadmium in their diet due to food uptake from contaminated soils.^[^ ^60^ ^]^ It is worth noting that the main cause of cadmium contamination of European agricultural soils is fertilization with phosphate fertilizers.^[^ ^61^ ^]^

The food supply chain is highly interconnected internationally, and disruptions have increasingly been of a transboundary nature.^[^ ^62^ ^]^ It is worth noting that the EU plays a significant role in international food markets. As 95% of our food is produced on soils, soil degradation, and health have a direct impact on food security and cross‐border food markets. Therefore, it is important to maintain soil health to ensure sustainable food production. An important consideration is that no EU country can claim to be entirely self‐sufficient when it comes to food security. Soil health has direct effects on crop yield, crop yield resiliency, and farmer profitability as well as affects global food security.^[^ ^63^ ^]^ The Global Food Security Index shows that the situation varies between Member States, but even the best‐performing EU countries still depend on soils beyond their borders and imports for the provision of food^[^ ^64^ ^]^.

Unhealthy soils have a negative impact on food production, which may affect food security both in the EU and globally. This is especially important considering the increasing global population and the EU's significant agri‐food export orientation. Soil health is directly related to food prices, as the balance between food supply and demand determines the price. It is worth noting that in 2021, four countries (Germany, France, Poland, and Romania) in the EU produced 60% of the cereals (EUROSTAT Data). Europe itself contributes to 34% of Global wheat production.^[^ ^65^ ^]^ Soil degradation in these countries may have a significant impact on the availability of agricultural products both domestically and internationally.

The agricultural sector in the EU experiences an estimated annual loss of 0.43% in crop productivity due to water erosion, which has been estimated to cost €1.25 billion.^[^ ^10^ ^]^ Furthermore, soil sealing has been found to result in potential loss of productive agricultural land. Between 1990 and 2006, soil sealing caused a loss of 0.81% of agricultural production in 19 EU countries, which is equivalent to 6 million tons of wheat.^[^ ^66^ ^]^ In addition, It has been observed that the use of heavy agricultural equipment in wet conditions leads to soil compaction, which in turn may result in a reduction of long‐term crop yields by 2.5–15%.^[^ ^67^ ^]^

**Figure 3:**
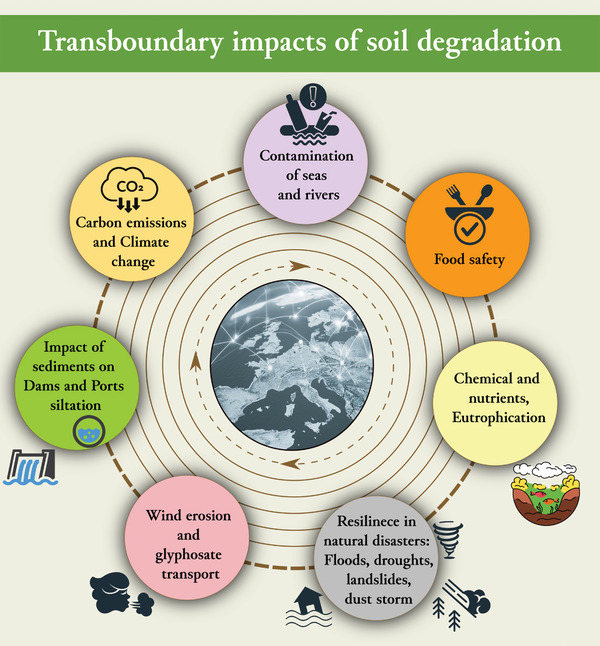
Examples of potential transboundary effects of soil degradation.

Close cooperation with EU neighbors and trading partners (countries trading food and feed with the EU) is as well necessary to address these cross‐border aspects of soil degradation (**Figure** **3**). However, it is important to address the issue within the EU first.

The transboundary effects of soil degradation reinforce the need for a pan European harmonized Soil Information system that will access soil degradation in a holistic way. Having in place a common monitoring framework will also reinforce the collaboration between regions and MS in order to remediate the negative transboundary effects. A good example is the Water Framework Directive which requires member states to cooperate with neighboring countries to achieve good ecological status for their shared water bodies.

## 6 Societal Challenges and Need for a Policy Framework at EU Level

Enhancing the accessibility of publicly available soil monitoring data is essential for increasing public awareness and fostering societal involvement in addressing soil‐related challenges. By sharing data and insights on soil health, stakeholders can make better‐informed decisions regarding sustainable soil management practices. This information can also play a crucial role in educating citizens about the importance of soil, aligning with the goals of the EU Mission ‘A Soil Deal for Europe’. Furthermore, soil monitoring efforts and the collected data can directly contribute to providing valuable information for ongoing research and development initiatives aimed at implementing measures to improve and sustain soil quality across the EU.

This intervention is crucial for ensuring food security,^[^ ^68^, ^69^ ^]^ as degraded soils become less productive due to climate change and intensive agriculture. Enhanced soil health will contribute to making EU food systems more resilient^[^ ^70^ ^]^ in the face of climate crises,^[^ ^71^ ^]^ unforeseen emergencies (e.g., the COVID‐19 pandemic), and geopolitical tensions (e.g., the Ukraine war). Soil degradation affects both farmers and consumers, as land productivity losses may drive up food prices and increase costs for farmers due to increased fertilizer inputs.

Furthermore, the Soil Monitoring Law will contribute to food safety by mitigating the status both in contaminated sites and in diffuse pollution. The free market rules in the EU allow free imports and exports of agricultural‐based food products inside the EU. Therefore, it is of huge importance to ensure for EU consumers that food production is safe (as it relates to soil health) and according to high health standards.

Climate change is an urgent issue that cannot be addressed solely at the MS level. The EU has proposed the Fit for 55 package and other initiatives, such as EU Carbon Removals and Carbon Farming Certification (CRCF), to contribute to climate change mitigation.^[^ ^7^ ^]^ It is therefore crucial to increase the current carbon concentrations in EU agricultural soils and reduce emissions from peatlands. In relation to natural disasters, droughts and floods are occurring more frequently and soils are becoming less resilient to those changes. Therefore, the impact of extreme events may affect the entire EU and not a single country.

The provision of ecosystem services by soils is a problem for individual countries and the entire Union, as soil degradation is not a local or regional issue. It has transboundary effects that must be addressed at the EU level, as mentioned earlier. The health of our soils is linked to the health of our ecosystems and the maintenance of below and above ground biodiversity. Soils are the cornerstone of one health and serve as a source and reservoir of pathogens, beneficial microorganisms, and the overall microbial diversity in a wide range of organisms (from microorganisms to large animals) and ecosystems.^[^ ^72^ ^]^

Finally, land degradation can distort the EU economy and its competitiveness, as workers operating in degraded lands are exposed to higher environmental risks and companies face higher costs in loans.^[^ ^73^ ^]^ A Soil Monitoring Law targeting healthy soils would contribute to increasing the legal and financial certainty of EU companies both in the EU single market and in the global economic environment.

## Conflict of interest

The authors declare no conflict of interest.
